# Understanding maternal mortality from top–down and bottom–up perspectives: Case of Tigray Region, Ethiopia

**DOI:** 10.7189/jogh.05.010404

**Published:** 2015-06

**Authors:** Hagos Godefay, Peter Byass, John Kinsman, Afework Mulugeta

**Affiliations:** 1 Tigray Regional Health Bureau, Mekelle, Ethiopia; 2 Umeå Centre for Global Health Research, Department of Public Health and Clinical Medicine, Umeå University, Umeå, Sweden; 3 Institute of Applied Health Sciences, School of Medicine and Dentistry, University of Aberdeen, Aberdeen, UK; 4 MRC/Wits Rural Public Health and Health Transitions Research Unit, School of Public Health, Faculty of Health Sciences, University of the Witwatersrand, Johannesburg, South Africa; 5 College of Health Sciences, Mekele University, Mekele, Ethiopia

## Abstract

**Background:**

Unacceptably high levels of preventable maternal mortality persist as a problem across sub–Saharan Africa and much of south Asia. Currently, local assessments of the magnitude of maternal mortality are not often made, so the best available information for health planning may come from global estimates and not reflect local circumstances.

**Methods:**

A community–based cross-sectional survey was designed to identify all live births together with all deaths among women aged 15–49 years retrospectively over a one–year period in six randomly selected districts of Tigray Region, northern Ethiopia. After birth and death identification, Health Extension Workers trained to use the WHO 2012 verbal autopsy (VA) tool visited households to carry out VAs on all deaths among women aged 15–49 years. All pregnancy–related deaths were identified after processing the VA material using the InterVA–4 model, which corresponds to the WHO 2012 VA. A maternal mortality ratio (MMR) was calculated for each District and expressed with a 95% confidence interval (CI).

**Results:**

The MMRs across the six sampled Districts ranged from 37 deaths per 100 000 live births (95% CI 1 to 207) to 482 deaths per 100 000 live births (95% CI 309 to 718). The overall MMR for Tigray Region was calculated at 266 deaths per 100 000 live births (95% CI 198 to 350). Direct obstetric causes accounted for 61% of all pregnancy–related deaths. Haemorrhage was the major cause of pregnancy–related death (34%). District–level MMRs were strongly inversely correlated with population density (r^2^ = 0.86).

**Conclusion:**

This simple but well–designed survey approach enabled estimation of maternal mortality in Tigray Region on a local, contemporary basis. It also provided insights into possible local variations in MMR and their determinants. Consequently, this approach could be implemented at regional level in other large sub–Saharan African countries, or at national level in smaller ones to monitor and evaluate maternal health service interventions.

Maternal mortality is one of the most sensitive indicators of the health disparities between poorer and richer nations, but also one of the most difficult health outcomes to measure reliably. Many estimation exercises and much debate have occurred around persistently unacceptable levels of maternal mortality in the world’s poorer countries, not least in relation to the fifth Millennium Development Goal (MDG5) [1]. However, in many settings major challenges remain in terms of both reducing and measuring maternal mortality effectively.

In an ideal world, all maternal deaths would be routinely registered, but in reality civil registration of deaths with cause is extremely scanty in sub–Saharan Africa and south Asia, where most maternal deaths occur [2]. Consequently global estimates from the UN agencies [3-5] and the Global Burden of Disease [6,7] have to apply very sophisticated modelling methods to these very scanty data in order to generate outputs that hopefully reflect realities of maternal mortality patterns, with varying degrees of success [8,9]. We characterize these processes here as “top–down”.

The alternative approach, for a country or a region, is to undertake direct measurement of maternal mortality, in order to inform health service management and planning, and to provide strategic insights in terms of necessary interventions. We characterize this as a “bottom–up” approach. Despite high top–down MMRs reported for Ethiopia [5,7], no current accurate estimate of the indicator and the underlying causes of avoidable maternal mortality is available on a population basis for the Tigray Region. This is partly due to the difficulties of finding and correctly identifying maternal deaths and ascertaining levels of maternal mortality at the community level. Hence local numbers for maternal deaths tend to only be derived from health facility data, which do not reflect the population–level situation. However, evidence on the magnitude and underlying causes of maternal deaths is essential for planning preventive measures to reduce maternal mortality in the Region. Accurate information on maternal deaths enables tracking progress of feasible health interventions, taking timely actions and increasing the intensity of accountability at all levels – government, civil society organizations, health care providers and donors [1,10-12].

Therefore, the aim of this study was to undertake a bottom–up assessment of maternal mortality for Tigray Region, in northern Ethiopia, identifying overall levels, specific causes and local determinants, and to compare the findings in relation to the various top–down estimates of maternal mortality that are available for Ethiopia.

## METHODS

### Study settings

**Figure 1:**
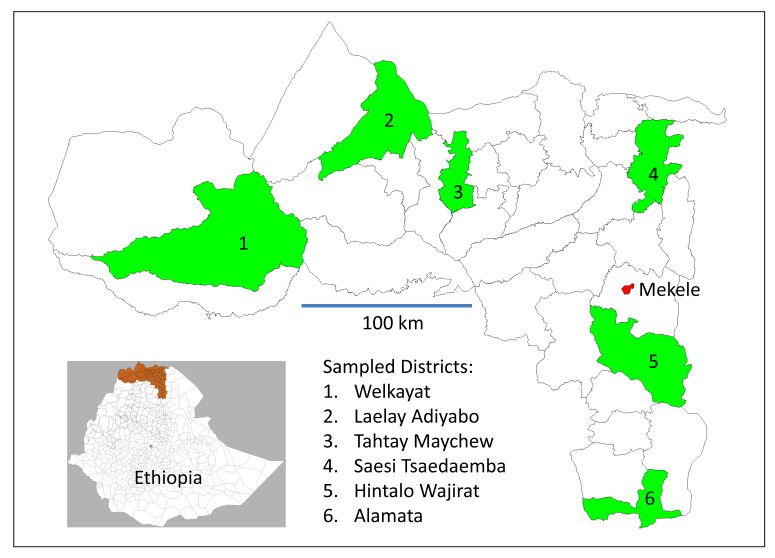
Location of study districts in Tigray regional state, northern Ethiopia (Source: Godefay et al. [13]).

Tigray Region is the northernmost of the nine Regional States of Ethiopia and has a total population of more than 5.1 million. The major urban centre is the regional capital, Mekele, from where health services are coordinated. Rural inhabitants constitute 81.5% of the Region’s population, living in an area of 50 078 km^2^, with a mean population density of 102 km^–2^. The maximum distances within the Region are 360 km east–west and 250 km north–south, and altitude above mean sea level varies between 600 m and 3950 m. The Region contains 1 165 575 households (HH), with an average of 4.4 persons per HH (3.4 persons per HH in urban areas and 4.6 persons per HH in rural areas). Health services in the Region are administered in six rural Zones, which are further divided into 34 Districts (locally known as *woreda*), each containing about 25 000 to 30 000 HHs (**Figure 1**). The Region is bordered to the north by Eritrea, to the south by Amhara Regional State, to the east by Afar Regional State and to the west by Sudan. In the Region, there are 15 hospitals, 214 health centres and 604 health posts [14]. Based on the Ethiopia Demographic and Health Survey 2011 (EDHS), conducted from September 2010 to June 2011, antenatal care coverage from a skilled provider was reported for 50.1% of pregnancies, and 11.6% of births were attended by skilled birth attendants, 0.9% by Health Extension Workers (HEWs) and 12.5% by trained Traditional Birth Attendants (TTBA) [14,15].

### Study design, sample and sampling procedure

Details of the study design and sampling process for this survey, accompanied by a parallel survey of under–5 deaths, have been described elsewhere [13]. Briefly, sample size estimates for the survey were calculated on the basis that a likely MMR might be 400/100 000 live births, which could be estimated within a 95% confidence interval of 300 to 522 if 54 maternal deaths were observed out of 13 500 live births. Assuming a crude birth rate of 30 per 1000, this would require a population base of 450 000. Since, for operational reasons, a sample clustered at the District level was required, a design factor of 2 led to target coverage of 900 000, approximately equivalent to six Districts.

The study was conducted in six rural Districts in Tigray region, namely Welkayat, Laelay Adiyabo, Tahtay Maychew, Saesi Tsaedaemba, Hintalo Wajirat and Alamata, which were randomly selected (using the Stata 12 *runiform* function to allocate a random number to every District, then selecting the District with the highest random number from each of the six Zones) as a stratified sample of one District per Zone, as shown in **Figure 1**. The sampled Districts included a total of 183 286 HHs, with a total population of 843 115, covering around 20% of the total population of rural Tigray. Of these, 166 515 were women of reproductive age, defined as 15–49 years, representing 19% of women of reproductive age in rural Tigray.

### Data collection procedure

**Figure 2:**
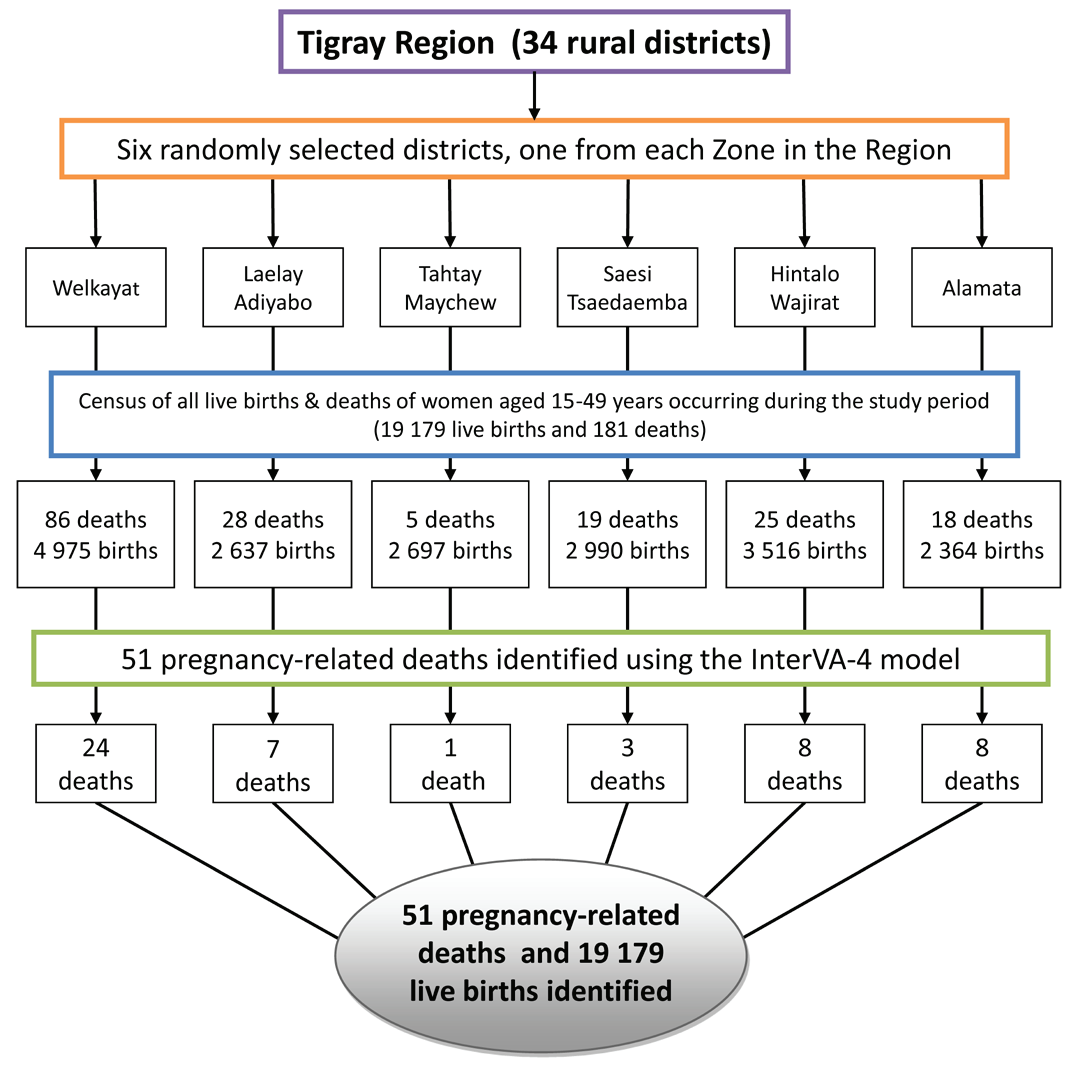
Schematic design of pregnancy–related mortality survey in six Districts, Tigray Region.

A census of all households was conducted in mid–2013 in the six randomly selected districts to identify all deaths to women of reproductive age that occurred between May 2012 and April 2013, irrespective of the cause, as shown schematically in **Figure 2**. In the same process all live births were identified. For each death identified in the selected districts, a trained health extension worker, responsible for all households in the sub district, visited the household of the deceased women to carry out a VA interview. Respondents were adult relatives who were caregivers at the time of death. The VA questionnaire used was adapted into the local language from the 2012 WHO Verbal Autopsy instrument for death of a person aged 15 years and above [16]. Supervisors were trained by the principal investigator for one day and the interviewers were trained by the supervisors for three days on the details of VA tool, interviewing techniques, indigenous terminologies, concepts of illnesses and their manifestations.

### Data processing and analysis

The VA data were processed using the InterVA–4 (version 4.02) model, using probabilistic modelling to assign cause of death instead of physicians [17,18]. This resulted in up to three probable causes of death per case, each with an associated likelihood. Cases where there was inadequate detail from the interview material – perhaps because of a lack of direct knowledge by the VA respondent – led to a totally indeterminate outcome, and where details were scanty the three probable causes did not achieve likelihoods summing to 100%. Thus residual likelihoods were assigned to be of indeterminate cause as recommended in the InterVA literature [18]. Consequently the indeterminate proportion of deaths encapsulates the degree of certainty with which cause of death assignments were possible. Finally, all deaths to women of reproductive age that occurred during the target year while pregnant or within 6 weeks of pregnancy ending or abortion, as classified by the InterVA–4 model, were considered as pregnancy related (**Figure 2**). As discussed below, we made the conservative assumption for calculating MMR to consider all pregnancy–related deaths as maternal deaths. The total number of pregnancy–related deaths identified through the VA and the total number of live births identified through the household census were used to compute maternal mortality ratios (MMR) and expressed per 100 000 live births. To calculate 95% confidence intervals around MMR estimates, the Poisson distribution was assumed, using the Stata 12 *cii* command.

### Ethics

Ethical approval for the study was granted by the Institutional Review Board (IRB) of the College of Health Sciences of Mekele University, Ethiopia.

## RESULTS

A total of 181 deaths among women of reproductive age and 19 179 live births were identified in the six selected Districts within the 12–month study period. Of the 181 deaths, 51 (28%) were ascertained as pregnancy–related deaths. **Table 1** summarizes the characteristics of the women who died of pregnancy related causes. Overall 24/51 (47.1%) of these deaths occurred in Welkayat district. The 25–34–year age group accounted for 23 (45.1%) of the deaths and 49 (96.1%) of the women who died were married or living with a partner. Forty one (80.4%) of the women who died had no formal education. Twenty eight (54.9%) of the deaths occurred after delivery or within six weeks of pregnancy ending and 23 (45.1%) of deaths occurred during pregnancy. Of the 28 women who died after delivery, 23 (82.1%) gave birth at home, while only 5 (17.9%) delivered at a health facility.

**Table 1:** Characteristics of 51 women who died during pregnancy or within 42 days of pregnancy ending in 6 sampled Districts, Tigray Region, Ethiopia, from March 2012 to April 2013

Characteristic	Number	%	Total population	Health centres	Health posts
Welkayat	24	47.1	171 302	7	25
Laelay Adiyabo	7	13.7	137 296	5	22
Tahtay Maychew	1	2.0	121 355	5	19
Saesi Tsaedaemba	3	5.9	122 479	7	26
Hintalo Wajirat	8	15.7	186 244	7	23
Alamata	8	15.7	104 439	5	15
**Age group (years):**					
15–24	10	19.6			
25–34	23	45.1			
35–49	18	35.3			
**Marital status (at death):**					
Never married	2	3.9			
Married or living with a partner	49	96.1			
**Education level:**					
No formal education	41	80.4			
Started formal education	10	19.6			
**Occupation:**					
Housewife	36	70.6			
Farmer	9	17.6			
Private employee	4	7.8			
Other	2	3.9			
**Place of death:**					
Health facility	15	29.4			
Home	36	70.6			
**Timing of death:**					
Pregnancy ended within 6 weeks of death	28	54.9			
Pregnant at death	23	45.1			
**Place of delivery (n = 28):**					
Health facility	5	17.9			
Home	23	82.1			

**Figure 3:**
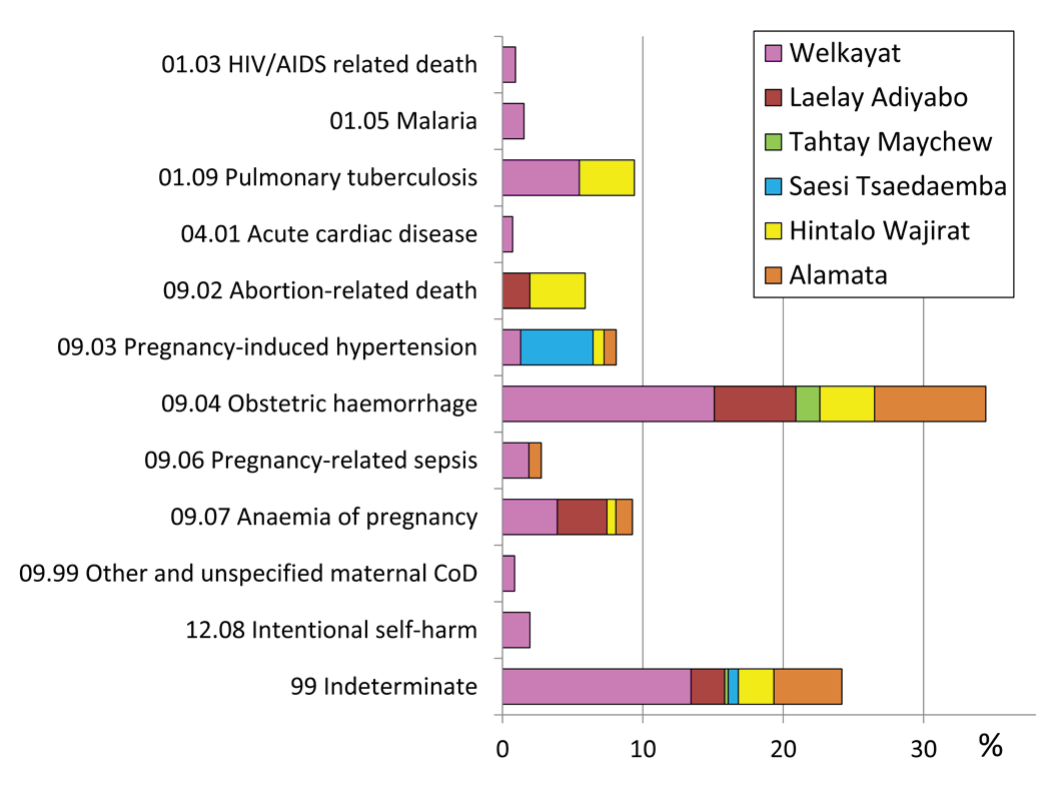
Percentage distribution of 51 pregnancy–related deaths among women aged 15–49 years from March 2012 to April 2013, Tigray Region, Ethiopia, by WHO verbal autopsy cause categories and District.

**Figure 3** shows causes of death in WHO 2012 VA cause of death categories (16) for the 51 pregnancy–related deaths, by District, as determined by the InterVA–4 model. In addition, the commonest causes of indirect causes of maternal death identified were anemia (12%) followed by pulmonary tuberculosis (10%), with both HIV/AIDS and malaria causing 2% of maternal deaths. Out of the 51 pregnancy–related deaths recorded, 61.3% were ascribed to direct obstetric causes. The most common obstetric causes were obstetric haemorrhage (34.4%), followed by anaemia of pregnancy (9.3%) and pregnancy–induced hypertension (8.1%). Post–abortion deaths accounted for 5.9% of pregnancy–related mortality.

**Figure 4:**
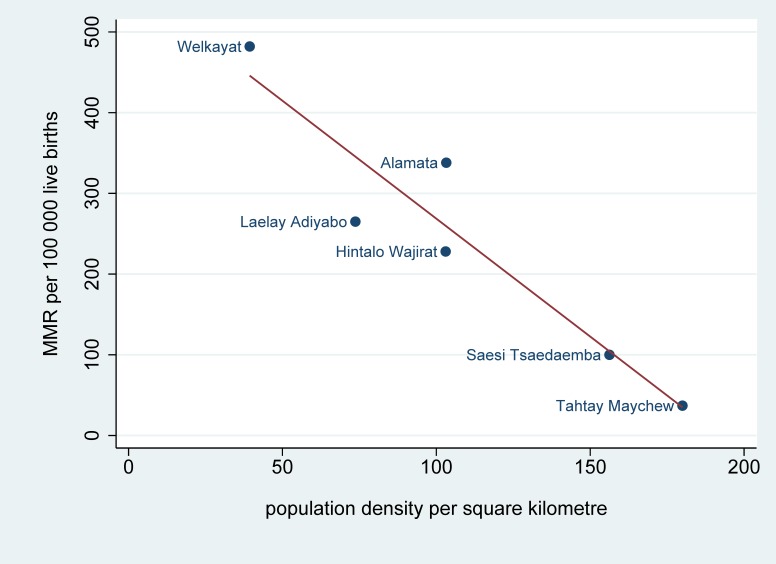
Correlation between maternal mortality ratios (MMR, per 100 000 live births) and population density (km^–2^) for six Districts in Tigray Region, Ethiopia (r^2^ = 0.86, *P* = 0.005).

**Table 2** shows estimates of MMR by District, age group, marital status and education. The magnitude of MMR ranged from 37 deaths per 100 000 live births (95% CI 1–207) in Tahtay Maychew District to 482 deaths per 100 000 live births (95% CI 309–718) in Welkayat District. Thus there were statistically significant variations in MMR within the six Districts surveyed. In view of the substantial variations in MMR between Districts, we looked at possible geographic determinants of these differences, including the populations and surface areas of Districts and distances from the Regional capital, Mekele. We found that MMR was significantly correlated with population density at the District level (r^2^ = 0.86, *P* = 0.005), as shown in **Figure 4**. MMR did not vary significantly by age group, marital status or education, though the small number of deaths among and births to unmarried women appeared to carry a higher risk. The overall estimate of MMR across all six Districts was 266 per 100 000 (95% CI 198–350).

**Table 2:** Maternal mortality ratios (MMR) per 100 000 live births among rural women aged 15–49 years in Tigray Region, Ethiopia, from March 2012 – April, 2013

Characteristic	Number of pregnancy–related deaths	Number of live births	MMR per 100 000 live births* (95% CI)
**District**			
Welkayat	24	4975	482 (309–718)
Laelay Adiyabo	7	2637	265 (107–547)
Tahtay Maychew	1	2697	37 (1–207)
Saesi Tsaedaemba	3	2990	100 (21–293)
Hintalo Wajirat	8	3516	228 (98–448)
Alamata	8	2364	338 (146–667)
**Age group (years):**			
15–24	10	3076	325 (156–598)
25–34	23	9432	244 (155–366)
35–49	18	6668	278 (165–440)
**Marital status:**			
Never married	2	247	810 (98–2925)
Married or living with a partner	49	18 300	268 (198–354)
**Education level:**			
No formal education	41	14 979	274 (196–371)
Started formal education	10	4110	243 (117–447)
**Overall**	51	19 179	266 (198–350)

CI – confidence interval

*Calculated using all pregnancy–related deaths.

**Figure 5:**
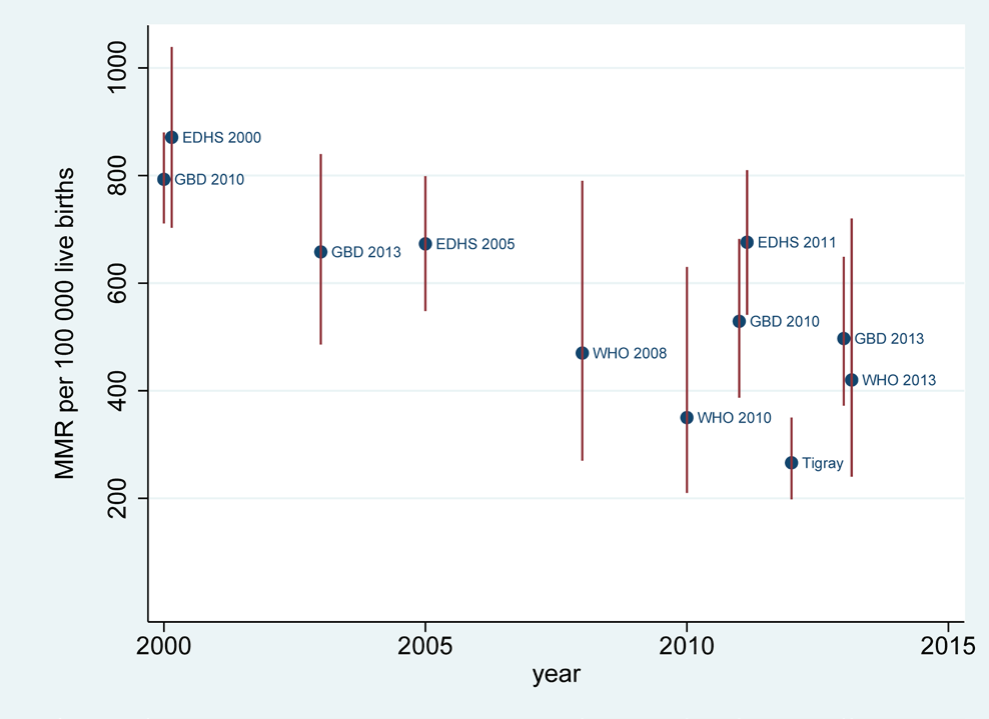
Available top–down estimates of maternal mortality ratios (MMR, per 100 000 live births) for Ethiopia made in recent years, together with the results for Tigray Region from this study, 2012–2013. (GBD 2013 [7]; EDHS 2005 [15]; WHO 2008 [3]; EDHS 2011 [15]; GBD 2010 [6]; WHO 2010 [4]; WHO 2013 [5]).

In contrast to these bottom–up results, **Figure 5** shows a compilation of available top–down estimates for MMR in Ethiopia in recent years [3-7,15].

## DISCUSSION

The major findings from this study were a new assessment of MMR for six districts in the Tigray Region at 266 deaths per 100 000 live births (95% CI 198–350), and an indication of possible wide variations between the sampled Districts. The confidence interval for the six districts’ MMR overlaps the uncertainty intervals for the UN national estimates for Ethiopia, which reported 350 deaths per 100 000 live births (uncertainty interval 210–630) for 2010 [4] and 420 (uncertainty interval 240–720) for 2013 [5]. The WHO national MMR estimates for neighbouring countries (Eritrea, Sudan and Djibouti) in 2013 also overlapped with our results from Tigray. Estimates for Ethiopia from the Global Burden of Disease project were generally higher, with smaller uncertainty intervals, than the WHO estimates, for reasons that are not entirely clear [6,7]. All of the global estimates models rely heavily on available data going back to at least 1990 to make their current estimates, and this tends to mean that the models are not very sensitive to rapid changes in MMR that may occur in recent periods, particularly if there is a lack of corresponding new data at the national level. Because other Regions do not have their own current estimates of MMR, it is impossible to know where these estimates from Tigray would rank in the national picture for Ethiopia.

Findings on MMR in this study were much lower than those from various rounds of the Ethiopian Demographic and Health Survey (EDHS), which reported an MMR of 676 per 100 000 live births (95% CI 541–810) for the period 2005–2011 [15], even though DHS uses the same definition of pregnancy–related deaths and MMR. This may be partly due to the EDHS methodology providing estimates for a six year period, and may reflect higher levels of MMR in some other Regions. Unfortunately the EDHS samples are not large enough to break down MMR by Region. Thus it was not possible to see how Tigray as a whole has performed in relation to MDG5.

Tigray Health Bureau has introduced a number of innovations targeted at reducing maternal mortality in recent years. These include leadership commitment and innovative approaches to supporting organized community mobilization for health. A woman–centred “one to five network” encourages every woman of reproductive age to engage voluntarily with small, local groups of neighbouring women (with 30 households on average being organized into a “Women’s Development Team”). Health facilities are being made more friendly to community members, preparing pregnant mothers for skilled delivery and post–natal care through monthly meetings, establishment of maternity waiting rooms, and offering traditional Ethiopian coffee ceremonies at delivery facilities. There is substantially increasing coverage of four–wheel drive ambulance services in every District, as well as “traditional ambulances” (locally made stretchers for carrying women in labour to health institutions in remote places). Additionally, one or two midwives trained in basic emergency management of neonatal care (BEMONC) have been assigned for every 25 000 population and basic emergency obstetric services like emergency surgery units placed in primary hospitals (designed to provide comprehensive preventive and curative service for every 100 000 population) are substantially more accessible than previously [19].

Our study showed that the majority of pregnancy–related deaths (36, 70.6%) occurred at home. This finding is comparable with studies conducted in India [20] and other low and middle income countries [21]. There may be many reasons for that, but it highlights the challenge to not only provide emergency obstetric services in an environment like Tigray, but also to get women in urgent need of care to facilities. Direct obstetric causes accounted for the majority (61.3%) of pregnancy–related deaths. The main direct and indirect causes were haemorrhage (34.4%), anaemia of pregnancy (9.3%) and pregnancy–induced hypertension (8.1%). These are all causes which can be reduced by effective emergency antenatal care and skilled care at birth to prevent, detect and manage mild complications, and obstetric care. However, our results were consistent with other studies, for example in Sokoto, Western Nigeria [22] which reported 48.3% of maternal deaths due to postpartum haemorrhage and a previous study from Tigray that reported 39% of maternal deaths due to haemorrhage [23]. Pregnancy–induced hypertension has also been reported as a major cause of maternal death in other studies, for example 19% in Nigeria [22], 28% in Haiti [24] and 19% in Tigray [11]. This might reflect a lack of access to anticonvulsant drugs like magnesium sulphate, either because of stock issues or lack of appropriate use, assuming that women get to facilities in the first place.

Indirect causes of maternal mortality are difficult to account for using verbal autopsy methods because of the need to make a judgment about the extent to which final illnesses might be ascribed to pregnancy. Garenne and colleagues have discussed the relevance of the concept of indirect maternal mortality, particularly in relation to the impact of HIV infection on pregnancy–related mortality [25]. Consequently, in this study we have taken the conservative approach of including all pregnancy–related deaths in estimates of MMR, even if that results in higher estimates of maternal deaths. This also avoids the difficulty of how to assign pregnancy–related deaths of indeterminate cause to maternal deaths, which is a problem that inevitably arises where VA respondents do not have sufficient knowledge of the case details.

The wide variations in MMR across the six Districts surveyed were somewhat surprising. Some of this variation might be accounted for by factors such as remoteness of some Districts, with hard–to–reach and scattered populations, which in turn might lead to limited access to health services, transport and other infrastructure. In Welkayat District, which recorded the highest MMR, five sub–districts (locally known as *kebeles*) did not have Health Extension Workers in place. The high turnover of health workers due to difficult living environments and less participation by development partners such as non–governmental organizations and faith–based organizations may lead to higher MMRs in remote areas. Maternal deaths were reported to be clustered in one district in another study conducted in Artibonite, rural Haiti [24]. Because of the extent of variation between Districts in our study, we looked for geographical factors that might help to understand the causes. We found a strikingly strong correlation between population density and MMR (**Figure 4**). The reasons for this are not immediately clear, but, since various levels of health services are normally provided on a per–population basis, rather than by size of geographical area, it is likely that access to health care is more challenging in Districts with lower population densities. This in turn suggests that logistic constraints probably persist as a major determinant of maternal mortality.

Possible limitations of our study include potential recall bias in identifying pregnancy–related deaths up to a year after they occurred, which would not happen if real–time death registration were in place. Nevertheless the recall demands over a one–year period are substantially less than those required in the EDHS methodology. However, pregnancy–related deaths are generally considered to be important and therefore memorable events within households, and it has been demonstrated that VAs can be administered reliably even after relatively long recall periods [26]. There is a further potential problem arising from the possibility of a household dissolving after the death of the mother as a key member, and consequently not being included in a retrospective survey. This probably occurs relatively rarely in the typical extended family structures in rural Ethiopia. Unlike some surveys of maternal mortality, we avoided the bias that can arise by identifying maternal deaths before determining cause of death, since we followed up all identified deaths among women of reproductive age.

In a one–off survey of this kind, it is not possible to assess the dynamics of MMR. However, by chance, one of the Districts surveyed here, Alamata, was also surveyed using a very similar methodology as part of a malaria treatment investigation in 2005–7 [27]. Live births were not counted in that survey, but 23 obstetric deaths were recorded over that two–year period in the Alamata District, compared with 5 obstetric deaths over a one–year period in this study.

## CONCLUSIONS

Our success in arriving at a bottom–up contemporary estimate of MMR for a sample of Tigray Region, as a result of undertaking a relatively straightforward population survey, is encouraging. At the same time, we were able to gain insights on apparently wide inter–District variations which are potentially important for planning maternal health services in the Region. Our experience suggests that it may be quite feasible for regions within larger sub–Saharan African countries, or for smaller countries at national level, to undertake similar surveys of local MMR, and we suggest that this approach should also be used more widely to monitor, supplement and strengthen maternal death surveillance and response (MDSR). The correlation we found between MMR and population density suggests that logistic factors remain a major determinant of maternal mortality in Tigray. The majority of pregnancy–related deaths, as determined using the WHO 2012 and InterVA–4 verbal autopsy tools arose from potentially preventable causes. This highlights the need to provide preventive and emergency obstetric care that is not only clinically effective but also accessible. The use of community mass media to increase the awareness of mothers about the advantages of skilled delivery services and involvement of male decision–makers regarding maternal health services also need to be given priority. Additionally, providing better incentives for health workers to stay in the more remote areas, and enhancing the ambulance system in those areas, should ensure a better and more sustained service.
